# Environmental stress impairs photoreceptor outer segment (POS) phagocytosis and degradation and induces autofluorescent material accumulation in hiPSC-RPE cells

**DOI:** 10.1038/s41420-019-0171-9

**Published:** 2019-05-16

**Authors:** Sonal Dalvi, Chad A. Galloway, Lauren Winschel, Ali Hashim, Celia Soto, Cynthia Tang, Leslie A. MacDonald, Ruchira Singh

**Affiliations:** 1 Department of Ophthalmology (Flaum Eye Institute), University of Rochester, Rochester, NY USA; 2 Department of Biomedical Genetics, University of Rochester, Rochester, NY USA; 3 UR Stem Cell and Regenerative Medicine Institute, Rochester, NY USA; 4 Center for Visual Science, University of Rochester, Rochester, NY USA; 5 Present Address: Department of Pathology and Lab Medicine, University of Rochester, Rochester, NY USA

**Keywords:** Stem cells, Pluripotent stem cells

## Abstract

Retinal pigment epithelium (RPE) cell dysfunction is central to the pathogenesis of age-related macular degeneration (AMD), a leading cause of adult blindness. Aging, the single biggest risk factor for AMD development, favors increase in RPE autofluorescent material due to accumulation of POS-digestion by-products through lysosomal dysfunction and impaired POS degradation. Apart from aging, environmental agents affect lysosomal function in multiple model systems and are implicated in AMD. Iron (Fe) overload and cigarette smoke exposure are the two environmental factors that are known to affect the lysosomal pathway and impact RPE cell health. However, the impact of Fe and cigarette smoke, on POS processing and its consequence for autofluorescent material accumulation in human RPE cells are yet to be established. Human induced pluripotent stem cell (hiPSC)-derived RPE, which phagocytoses and degrades POS in culture and can be derived from control individuals (no history/susceptibility for retinal disease), provides a model system to investigate the singular effect of excess Fe and/or cigarette smoke on POS processing by RPE cells. Using at least three distinct control hiPSC lines, we show that, compared to untreated hiPSC-RPE cells, POS uptake is reduced in both Fe (ferric ammonium citrate or FAC) and FAC + CSE (cigarette smoke extract)-treated hiPSC-RPE cells. Furthermore, exposure of hiPSC-RPE cultures to FAC + CSE leads to reduced levels of active cathepsin-D (CTSD), a lysosomal enzyme involved in POS processing, and causes delayed degradation of POS. Notably, delayed degradation of POS over time (2 weeks) in hiPSC-RPE cells exposed to Fe and CSE was sufficient to increase autofluorescent material build-up in these cells. Given that inefficient POS processing-mediated autofluorescent material accumulation in RPE cells has already been linked to AMD development, our results implicate a causative role of environmental agents, like Fe and cigarette smoke, in AMD.

## Introduction

Clinical and basic science research has revealed a significant role of environmental agents in the etiology of macular degeneration (MD). In particular, twin studies have shown that inheritance of MD ranges from 45% for early maculopathy to 71% for advanced age-related macular degeneration (AMD), supporting a genetic component but also emphasizing the role of environmental factors^1^. Two prominent environmental agents linked to AMD through epidemiological and experimental studies are excess iron (Fe) and cigarette smoke^2–8^. Interestingly, aging, a necessary requirement for AMD development, has been associated with increased Fe levels in both the mouse and human/retina^6,9,10^. Furthermore, cadaver eyes from patients with AMD show increased Fe deposits in retinal pigment epithelium (RPE) cells^11^. Importantly, mutations in *CP gene* lead to increased Fe in RPE cells/retina and cause maculopathy-like features in patients with aceruloplasminemia^12,13^. In addition, targeting Fe homeostasis through genetic ablation in rodent models has been shown to cause local Fe accumulation within RPE cells and maculopathy-relevant cellular changes^14–16^. Similarly, exposure risk for cigarette smoke, a prominent modifiable risk factor contributing to AMD^2,3,17,18^, is higher in adults aged 18–64^19^. Furthermore, chronic exposure to cigarette smoke in mice results in pathological alterations consistent with AMD^4^. Likewise, acute exposure of ARPE-19 cells, primary human RPE, and human fetal RPE to cigarette smoke extract (CSE) and/or toxic components of cigarette smoke such as [B(a)P] and acrolein leads to cellular alterations consistent with AMD (e.g., oxidative stress, increased autophagy, and cell death)^5,20,21^.

The accumulation of autofluorescent material (lipofuscin), metabolic debris from incomplete photoreceptor outer segment (POS) digestion, has been linked to AMD development through several plausible mechanisms, reduction in RPE cytoplasmic volume^22^, complement activation^23^, and RPE cell death^24^. In fact, aging, the biggest risk factor for AMD development, leads to a significant increase in RPE lipofuscin accumulation, with ~1% of the RPE cytoplasmic volume covered by lipofuscin in the first decade of life compared to ~19% by the age 80^25^. Interestingly, increased autofluorescent material accumulation in the RPE cells and RPE Fe overload have been reported to coexist in patients with aceruloplasminemia^12,26^. Furthermore, excess Fe in cells has been shown to selectively accumulate in lysosomes as a component of Fe-rich lipofuscin^27,28^. In fact, in ARPE-19 cells, excess Fe has been shown to alter the activity of cathepsin-D (CTSD)^6^, a lysosomal enzyme involved in degradation of POS^29,30^. Similarly, cigarette smoke has been linked to lysosomal dysfunction^31,32^ and altered CTSD activity in ARPE-19 cells and a murine model exposed to [B(a)P]^32^. Although these data indicate that like aging, cigarette smoke and Fe can influence POS processing, the impact of Fe and cigarette smoke on POS phagocytosis and degradation, and its consequence for accumulation of autofluorescent POS-digestion by-products, a pathological feature of AMD, have not been established in human RPE cells.

Human induced pluripotent stem cell (hiPSC) technology has provided a suitable platform to gain fundamental insights into several RPE-based disorders, including AMD and related MDs. For instance, hiPSC-RPE derived from patients with AMD and related macular dystrophies, Sorsby’s fundus dystrophy (SFD) and Doyne honeycomb retinal dystrophy, have shown the ability to mimic both disease-associated molecular alterations with complement pathway alteration^33,34^ and pathological changes such as drusen formation and extracellular matrix protein accumulation^34,35^. Notably, disease modeling efforts using hiPSC-RPE-derived cell models have utilized the unique ability to select a specific patient population to investigate the (i) precise impact of genetic defects on monogenic diseases with complete penetrance [e.g., best disease (BD)^36,37^, SFD^34^, and *MERTK* mutations in Retinitis pigmentosa (RP)^38,39^], as well as the (ii) consequence of a specific protective/risk haplotype in individual genes (e.g., *CFH*^35^) in a complex multifactorial disease like AMD. Although genetic defects have been central to disease modeling efforts using hiPSC-RPE cells, it is plausible that hiPSC-RPE from healthy individuals with no history of retinal disease provides a suitable platform to interrogate the singular role of environmental agents in the development of disease-relevant molecular and pathological changes in human RPE cells. This is especially pertinent to cellular process(es), such as phagocytosis and degradation of POS, and linked pathological changes, such as increased accumulation of autofluorescent material, that can be faithfully mimicked in an hiPSC-RPE model system.

In this study, we utilized hiPSC-RPE from at least three distinct control hiPSC lines, with no known genetic susceptibility for AMD development, to evaluate the singular impact of cigarette smoke (CSE, 0.5%/day) and/or Fe (ferric ammonium citrate or FAC, 50 and 200 µg/ml/day) on (i) lysosomal function and POS processing and (ii) its consequence for autofluorescent material accumulation in RPE cells.

## Results

### Control hiPSC-RPE lines used in this study display key morphological, protein expression, and functional attributes of RPE cell in vivo

**Fig. 1:**
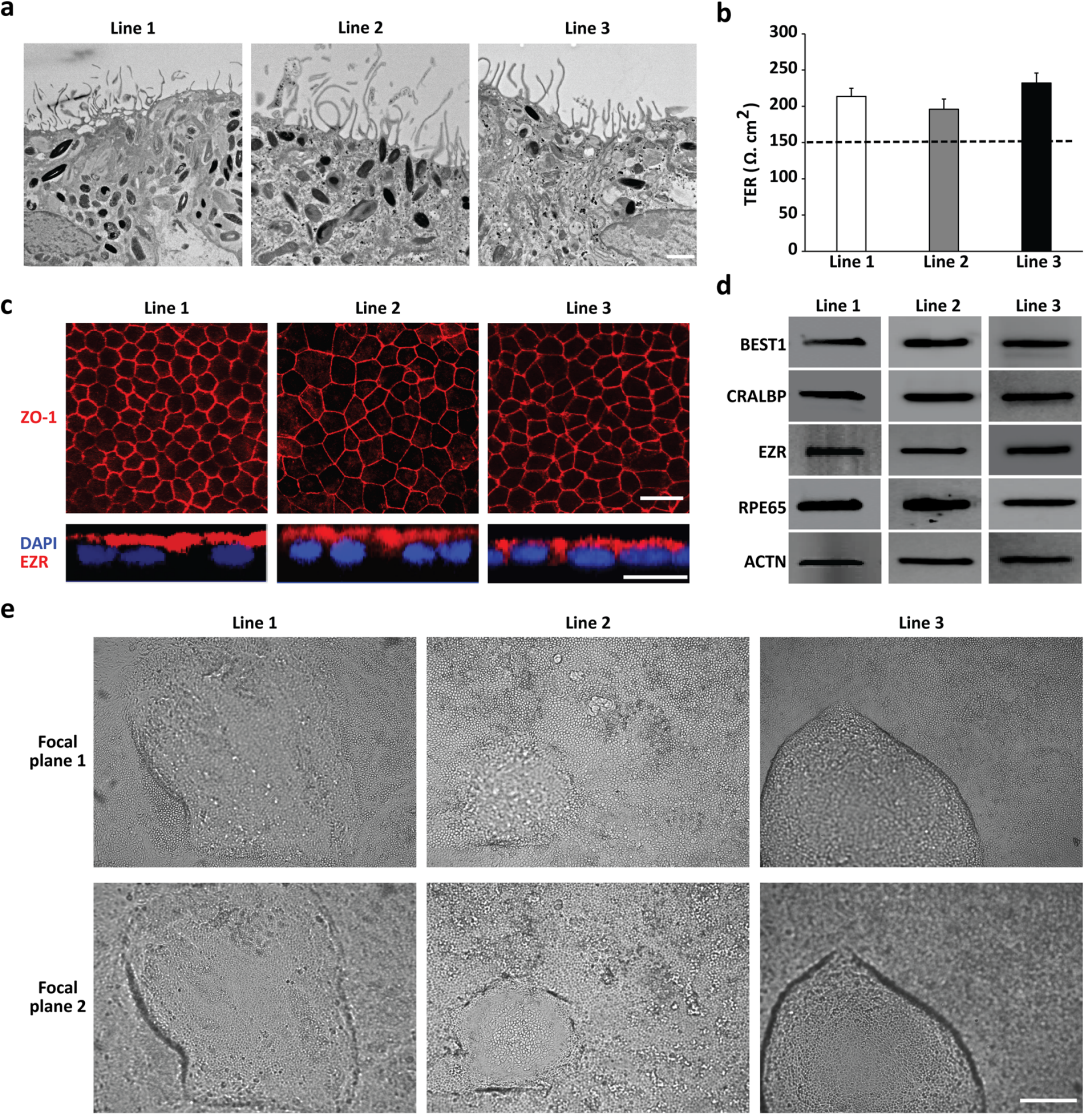
Control hiPSC-RPE lines utilized in this study display important cellular characteristics of human RPE cells in vivo. **a** Representative electron microscopy images depicting the presence of RPE-characteristic microvilli structures and apically migrating melanosomes in all hiPSC-RPE lines, lines 1–3 (scale bar = 1 µm). **b** Graphical representation of transepithelial resistance (TER) measurements showing TER ≥ 150 Ω cm^2^ (dotted line; the reported in vivo TER threshold^40^) in all hiPSC-RPE lines. Data are represented as mean ± SEM. **c** Representative confocal images displaying the expected localization of tight junction marker, ZO-1, and apically expressed RPE protein, EZR, in all hiPSC-RPE lines (scale bar = 20 µm). **d** Representative western blot images showing robust expression of several RPE signature proteins (BEST1, CRALBP, EZR, and RPE65) in all hiPSC-RPE lines. Of note, ACTN served as a loading control for western blot analyses. **e** Light microscopy images showing the presence of fluid domes, a characteristic of polarized epithelium, in hiPSC-RPE lines grown on nonporous tissue culture plastic support. Of note, the RPE morphology in focal plane 1 can be seen in the RPE monolayer outside the fluid dome, whereas the RPE cells within the fluid dome can be clearly seen in focal plane 2 (scale bar = 250 µm)

Electron microscopy analyses of hiPSC-RPE lines utilized in this study consistently displayed typical RPE features, including microvilli and apically migrating melanosomes (Fig. 1a). Furthermore, transepithelial resistance (TER) measurements of hiPSC-RPE cultures derived from distinct hiPSC lines showed the presence of functional tight junctions with (TER) ≥ 150 Ω cm^2^, an in vivo reported threshold of TER^40^ (Fig. 1b). Immunocytochemical analyses of hiPSC-RPE lines further demonstrated expected pattern and robust expression of tight junction marker, ZO-1, (Fig. 1c) and apically localized RPE protein, EZR (Fig. 1c). Additionally, western blot analyses revealed strong and similar expression of several RPE signature proteins, BEST1, CRALBP, EZR, and RPE65, in the hiPSC-RPE lines (Fig. 1d). Of note, ACTN served as a loading control for western blot analyses experiments (Fig. 1d). Lastly, consistent with the formation of a polarized RPE monolayer, when hiPSC-RPE cell lines were grown on nonpermeable plastic support, they displayed the presence of fluid domes^34,36^ (Fig. 1e).

### Acute exposure to excess Fe (FAC) is sufficient to impair POS phagocytosis by hiPSC-RPE cells

**Fig. 2:**
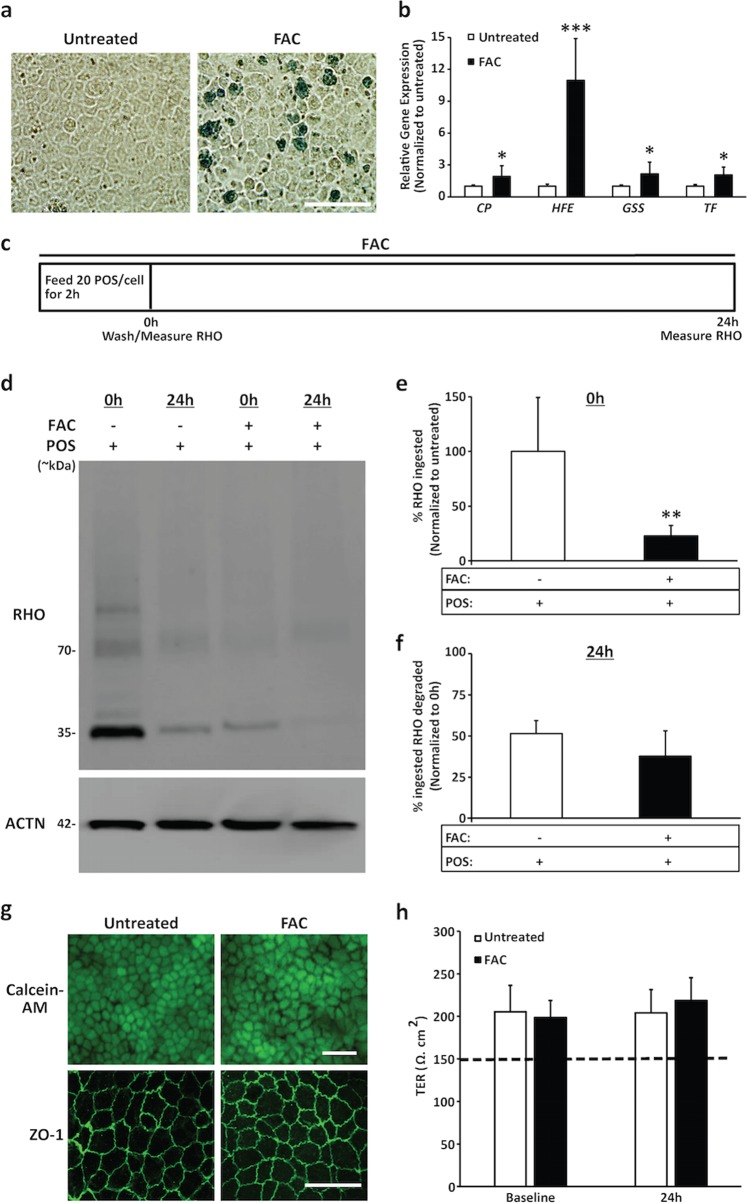
FAC supplementation of culture media is sufficient to alter iron (Fe) metabolism and impair POS uptake by hiPSC-RPE cells. **a** Representative images of parallel cultures of Prussian blue-stained untreated and FAC-treated (50 µg/ml, ~1 month) hiPSC-RPE cells showing increased presence of Fe deposits in FAC-treated hiPSC-RPE cells (scale bar = 50 µm). **b** Quantitative analyses of real-time PCR demonstrating increased expression of iron-regulatory genes (*CP, HFE, GSS*, and *TF*) in untreated versus FAC-treated (50 µg/ml, ~1 month) hiPSC-RPE cultures. Data are represented as mean ± SEM. *P ≤ 0.05, ***P ≤ 0.001, *n* = 3 independent trials. **c** A schematic depicting the protocol utilized for assessing the impact of acute FAC exposure (200 µg/ml, 24 h) on POS uptake (0 h time point) and degradation (24 h time point) post 2 h POS feeding (20 POS/RPE cell)^36^. Of note, the amount of POS in hiPSC-RPE cells was evaluated indirectly by measuring the protein levels of RHO, a POS-specific protein. **d**–**f** Representative western blot images **(d)** and corresponding quantitative analyses **(e**, **f)** showing reduced uptake of RHO/POS at the 0 h time point **(d**, **e)** but similar degradation of ingested RHO at the 24 h time point **(d**, **f)** in untreated versus FAC-treated hiPSC-RPE cultures. Data are presented as mean ± SEM. **P ≤ 0.01, *n* = 3 independent trials. Of note, to represent the rate of POS degradation, the quantification of RHO levels at 24 h time point is calculated relative to the amount of POS uptake (0 h time point). Note: ACTN served as loading control for western blot analyses. **g** Representative fluorescent microscopy (top panel) and confocal microscopy images (bottom panel) showing no difference in cell viability (calcein-AM) and localization of tight junction marker, ZO-1, in untreated versus FAC-treated (200 µg/ml, 24 h) hiPSC-RPE cultures (scale bar = 20 µm). **h** Quantitative analyses of TER measurements showing similar TER values with TER ≥ 150 Ω cm^2^ (dotted line; the reported in vivo TER threshold^40^) at baseline and the 24 h time point in both untreated and FAC-treated (200 µg/ml, 24 h) hiPSC-RPE cultures. Data are presented as mean ± SEM. *n* = 3 independent trials

Given that the amount of FAC (50–200 µg/ml)^41–44^ utilized in this study to assess the acute (24 h) and chronic (~2 weeks to 1 month) impact of excess Fe was based on previously published studies in primary RPE cell cultures (mouse and human fetal RPE) and immortalized cell lines (HepG2 and ARPE-19), we first confirmed that FAC exposure was sufficient to elicit the expected (i) intracellular accumulation of Fe deposits (Fig. 2a) and (ii) altered expression of Fe-regulatory genes (*CP, HFE, GSS*, and *TF*) (Fig. 2b, Supplementary Table 1) in FAC-treated hiPSC-RPE cultures. Next, to determine the impact of excess Fe on POS phagocytosis and degradation, hiPSC-RPE cells were simultaneously fed POS (20 POS/RPE cell) and exposed to FAC (Fig. 2c). POS uptake (2 h post-POS feeding; 0 h time point) and degradation (24 h post-POS feeding; 24 h time point) were then determined by quantification of RHO, a POS-specific protein (Fig. 2c). With regard to RHO quantification, the cumulative amount of RHO, including monomer, dimer, and multimers^36^ and potentially the post-translationally modified forms (e.g., glycosylated RHO)^45^ relative to loading control ACTN, was utilized to assess the amount of RHO/POS in hiPSC-RPE cultures. Of note, parallel cultures of hiPSC-RPE fed with only POS served as untreated controls in these experiments. Consistent with a prior study in the ARPE-19 cell line that showed reduced POS phagocytosis in the presence of excess Fe^6^, quantitative western blot analyses of FAC-treated versus untreated hiPSC-RPE cultures revealed lower levels of RHO/POS in the FAC-treated hiPSC-RPE cells (22.82 ± 9.42%) at the 0 h time point compared to the untreated hiPSC-RPE cells (100 ± 49.37%, *P* ≤ 0.01) (Fig. 2d, e). In contrast, no difference in the rate of RHO/POS degradation, the amount of ingested POS degraded within 24 h, was observed between untreated (51.42 ± 7.96%) and FAC-treated (37.68 ± 15.49%) hiPSC-RPE cultures (Fig. 2d, f). Notably, acute exposure to FAC did not adversely impact cell viability (Fig. 2g, top panel) or morphology (Supplementary Fig. 1) of hiPSC-RPE cultures. Furthermore, similar localization of tight junction marker, ZO-1 (Fig. 2g, bottom panel), and TER measurements (Fig. 2h) were seen in untreated versus FAC-treated hiPSC-RPE cultures. Altogether, these results showed that FAC supplementation of cell culture media can alter Fe homeostasis and cause Fe overload in hiPSC-RPE cultures. Furthermore, nontoxic levels of excess Fe by itself in the absence of a specific gene defect is sufficient to impair POS uptake in hiPSC-RPE cells.

### Acute exposure to cigarette smoke extract (CSE) has no effect on POS phagocytosis and degradation by hiPSC-RPE cells

**Fig. 3:**
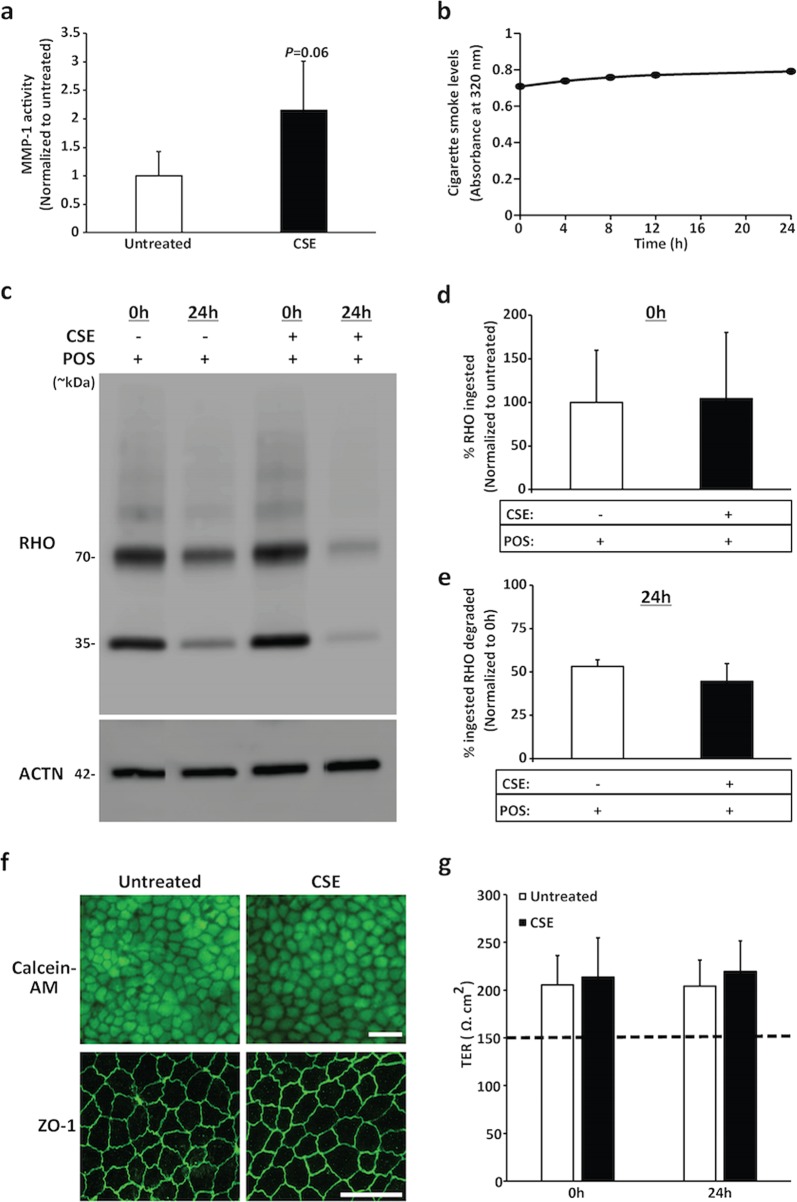
CSE supplementation of culture media is sufficient to alter MMP-1 activity but has no impact on POS phagocytosis and degradation by hiPSC-RPE cells. **a** Zymography analyses of basally secreted media from parallel cultures of untreated versus CSE-treated (0.5%, 2 weeks) hiPSC-RPE cultures showed increased MMP-1 activity in CSE-treated hiPSC-RPE cultures. Data are presented as mean ± SEM, *P* = 0.06, *n* = 3 independent trials. **b** Spectrometric measurement of cell culture media at distinct time points (0 h, 4 h, 8 h, 12 h, and 24 h) during a 24 h period after addition of CSE (0 h) to the media showed constant cigarette smoke level as measured by absorbance at 320 nm^71^. **c–e** Representative western blot images (**c**) and corresponding quantitative analyses (**d**, **e**) showing similar uptake (0 h) (**c**, **d**) and degradation (24 h) (**c**, **e**) of RHO/POS in untreated versus CSE-treated (0.5%) hiPSC-RPE cultures. Data are presented as mean ± SEM, *n* = 5 independent trials. Note: ACTN served as loading control for western blot analyses. **f** Representative fluorescent microscopy (top panel) and confocal microscopy images (bottom panel) displaying similar cell viability (calcein-AM) and ZO-1 localization in untreated versus CSE-treated (0.5%, 24 h) hiPSC-RPE cultures (scale bars = 20 µm). **g** TER measurements of untreated versus CSE-treated (0.5%, 24 h) hiPSC-RPE were similar at baseline and the 24 h time point with TER ≥ 150 Ω cm^2^ (dotted line, the reported in vivo TER threshold^40^). Data are presented as mean ± SEM. *n* = 3 independent trials

CSE at a concentration of 0.5%, a dose that has previously been shown to affect ARPE-19 cell health and function^5^, was used to assess the effect of cigarette smoke on POS phagocytosis and degradation by hiPSC-RPE cells. Importantly, consistent with previously described regulation of MMP-1 activity by cigarette smoke^46^, chronic exposure (2 weeks) of hiPSC-RPE cultures to CSE led to increased activity of secreted MMP-1 (Fig. 3a). Furthermore, hiPSC-RPE cultures supplemented with CSE (0.5%)^5^ showed constant levels of CSE, as measured by absorbance at 320 nm, for the 24 h period of daily CSE treatment (Fig. 3b). To evaluate the effect of cigarette smoke on POS phagocytosis and degradation by hiPSC-RPE cells, we utilized acute (24 h) CSE exposure as the extrinsic stressor in the previously described phagocytosis and degradation experiment (Fig. 2c). In contrast to FAC-treated RPE cells (Fig. 2d–f), western blot analyses of untreated versus CSE-treated hiPSC-RPE cells showed no difference in RHO/POS abundance at the 0 h time point (CSE-treated: 104.21 ± 75.8% versus untreated: 100 ± 59.78%, *P* = 0.55) (Fig. 3c, d). Furthermore, untreated and CSE-treated hiPSC-RPE cells degraded a similar amount of RHO at the 24 h time point (CSE-treated: 44.62 ± 10.26% versus untreated: 53.17 ± 3.94%, *P* = 0.51) (Fig. 3c, e). In addition, CSE exposure (24 h) did not impact the cell viability (Fig. 3f, top panel), morphology (Supplementary Fig. 1), ZO-1 localization (Fig. 3f, bottom panel), and TER (Fig. 3g) in hiPSC-RPE cultures. Taken together, these results show that although supplementation of hiPSC-RPE cultures with a nontoxic dose of CSE can elicit the documented smoke-induced change in MMP-1 activity^46^, CSE exposure by itself is not sufficient to adversely affect POS phagocytosis and degradation.

### Combined exposure to FAC and CSE affects active-CTSD levels and impairs both POS phagocytosis and degradation in hiPSC-RPE cells

Although several studies have evaluated the impact of Fe or cigarette smoke exposure on RPE cell health in vitro and in vivo^4–6,14,16,47^, the impact of combined exposure on Fe and CSE together has not been evaluated. Simultaneous presence of iron overload in RPE cells and cigarette smoke exposure is a plausible scenario given the fact that aging has been shown to increase the levels of Fe in RPE cells^9^ and cigarette smoke is a significant risk factor linked to adult population^19^. Furthermore, given that lifestyle and dietary habits have been implicated in both smoke exposure (smoking cigarettes)^2^ and iron overload (eating red meat^48^) in AMD, it is not unlikely that excess Fe and cigarette smoke exposure together occur in a subset of people in the general population. Additionally, RPE iron overload and cigarette smoke exposure can occur together in patients with hemochromatosis who have iron overload in RPE cells^12^.

**Fig. 4:**
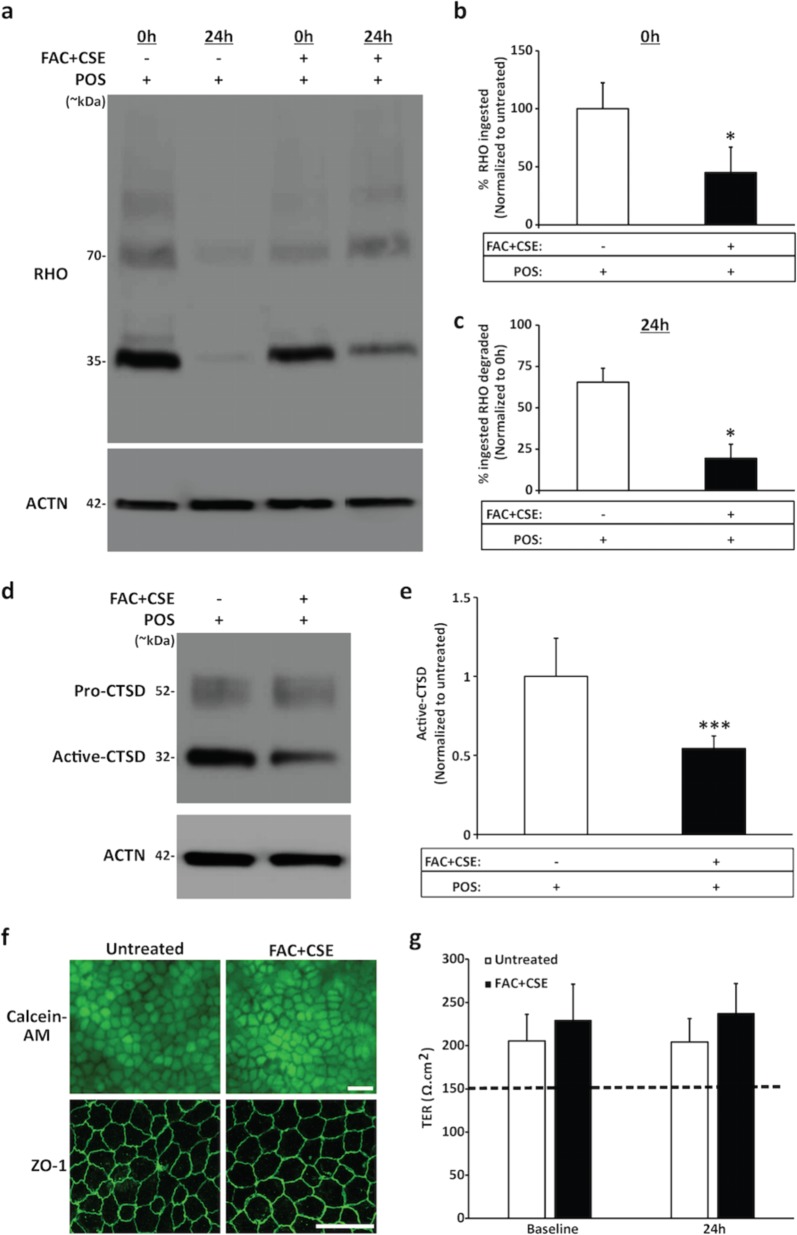
Combined supplementation of FAC and CSE to the culture media is sufficient to decrease both uptake and degradation of POS by hiPSC-RPE cells. **a–c** Representative western blot images (**a**) and corresponding quantitative analyses (**b**, **c**) showing reduced uptake of RHO/POS at the 0 h time point (**a**, **b**) and decreased degradation of ingested RHO/POS at 24 h time point (**a**, **c**) in untreated versus FAC + CSE-treated (200 µg/ml + 0.5%) hiPSC-RPE cultures. Data are presented as mean ± SEM, *n* = 3 independent trials. *P ≤ 0.05. ACTN served as loading control. **d–e** Representative western blot images (**d**) and corresponding quantitative analyses (**e**) displaying reduced levels of active CTSD (32 kDa) in POS-fed and FAC + CSE-treated hiPSC-RPE cultures compared to only POS-fed hiPSC-RPE cultures. Data are presented as mean ± SEM, *n* = 3 independent trials. *P ≤ 0.05, ***P ≤ 0.001. Note: ACTN served as loading control for western blot analyses. **f** Representative fluorescent microscopy (top panel) and confocal microscopy images (bottom panel) demonstrating equivalent staining of calcein-AM-positive live cells (top panel) and ZO-1 localization (bottom panel) in untreated and FAC + CSE-treated (200 µg/ml + 0.5%, 24 h) hiPSC-RPE cultures (scale bar = 20 µm). **g** TER ≥ 150 Ω cm^2^ was seen at both baseline and 24 h time point in untreated and FAC + CSE-treated (200 µg/ml + 0.5%, 24 h) hiPSC-RPE cultures. Data are presented as mean ± SEM. *n* = 3 independent trials

Consistent with the reduced POS uptake in FAC-treated cultures (Fig. 2d, e), FAC + CSE-treated hiPSC-RPE cells showed decreased amount of RHO at the 0 h time point when compared to untreated hiPSC-RPE cells (FAC + CSE-treated: 44.92 ± 22.04% versus untreated: 100 ± 22.41%, *P* ≤ 0.05, Fig. 4a, b). Furthermore, combined FAC and CSE treatment led to a decreased rate of RHO/POS degradation at the 24 h time point (FAC + CSE-treated: 19.46 ± 8.54% versus untreated: 65.49 ± 8.39%, *P* ≤ 0.05, Fig. 4a, c). A plausible role of impaired lysosomal function in decreased rate of POS degradation observed in FAC + CSE-treated cultures, was implied by the negative impact of FAC + CSE treatment on active-CTSD levels (Fig. 4d, e). Of note, CTSD is an integral lysosomal enzyme involved in POS degradation^29,30,49^. Specifically, slower POS degradation in FAC + CSE-treated cultures correlated with reduced active-CTSD levels (FAC + CSE-treated: 0.54 ± 0.08 versus untreated: 1 ± 0.23, *P* ≤ 0.001, Fig. 4d, e). In contrast, no change in the expression of pro-CTSD was seen between untreated and FAC + CSE-treated hiPSC-RPE cultures (FAC + CSE-treated: 0.98 ± 0.41 versus untreated: 1 ± 35, *P* = 0.76, Supplementary Fig. 2). To further confirm the correlation of reduced active-CTSD abundance with decreased POS degradation, we also determined the effect of FAC supplementation, a stressor that reduced POS uptake but not degradation, on active-CTSD levels in hiPSC-RPE cultures. Consistent with similar rate of POS digestion (Fig. 2d, f), untreated versus FAC-treated cultures showed similar expression of active-CTSD (FAC-treated: 0.96 ± 0.11 versus untreated: 1 ± 0.18, *P* = 0.76) (Supplementary Fig. 3). Notably, similar to FAC and CSE treatment, acute exposure (24 h) of hiPSC-RPE to FAC + CSE had no adverse effects on cell viability (Fig. 4f, top panel), RPE morphology (Supplementary Fig. 1), ZO-1 localization (Fig. 4f, bottom panel), and TER values (untreated: 229.13 ± 41.9, FAC + CSE treated: 237.16 ± 34.86, *P* = 0.58) (Fig. 4g). Altogether, these results show that combined exposure to excess Fe and cigarette smoke is sufficient to reduce the levels of active-CTSD, a POS processing lysosomal enzyme, and negatively impact POS degradation by hiPSC-RPE cells.

### Chronic exposure to FAC and CSE is sufficient to induce increased autofluorescent material accumulation in hiPSC-RPE cells

**Fig. 5:**
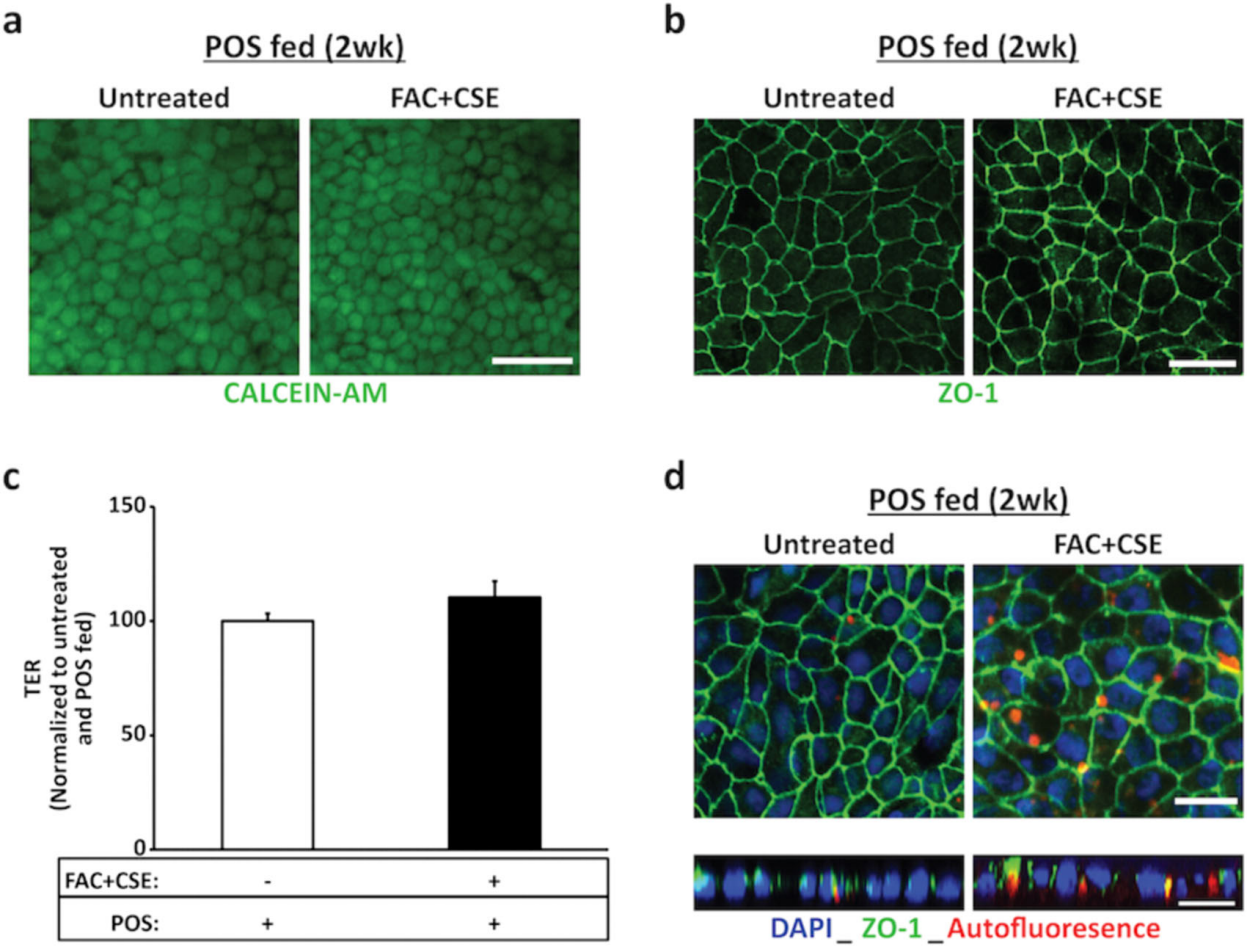
FAC + CSE supplementation of culture media over 2 weeks leads to intracellular accumulation of autofluorescent material in daily POS-fed hiPSC-RPE cultures. **a** Representative fluorescence microscopy images showing similar cell viability (calcein-AM) in FAC + CSE-treated (200 µg/ml + 0.5%) and POS-fed (20 POS/RPE/day) and untreated but POS-fed (20 POS/RPE/day) hiPSC-RPE cultures after 2 weeks of daily POS feeding and FAC + CSE supplementation to the culture media. Of note, untreated cultures had media change with regular cell culture media during daily POS feeding (scale bar = 25 µm). **b** Representative confocal images depicting similar tight junction morphology (ZO-1) in untreated but POS-fed (daily for 2 weeks) and FAC + CSE-treated (200 µg/ml + 0.5%, daily for 2 weeks) and POS-fed (20 POS/RPE/day for 2 weeks) hiPSC-RPE cultures. (Scale bar = 25 µm). **c** TER measurements showing similar TER values in POS-fed and FAC + CSE-treated (200 µg/ml + 0.5%) and untreated but POS-fed hiPSC-RPE cultures after 2 weeks of daily POS feeding and FAC + CSE supplementation. Data are presented as mean ± SEM, *n* = 3 independent trials. **d** Representative confocal microscopy images at the 2 weeks time point showing increased accumulation of autofluorescent material in spectral wavelength (ex: 546 nm, em: 560–615 nm) consistent with lipofuscin accumulation (top panel) in FAC + CSE-treated and POS-fed hiPSC-RPE cultures compared to untreated but POS-fed hiPSC-RPE cultures. Furthermore, orthogonal view of hiPSC-RPE cultures comparing the localization of autofluorescence accumulation (red) in relationship to tight junction marker ZO-1 (green) and Hoechst-stained cell nuclei (blue) confirming the intracellular localization of autofluorescent particles in both untreated and FAC + CSE-treated POS-fed cultures (bottom panel). (Scale bar = 25 µm)

**Fig. 6:**
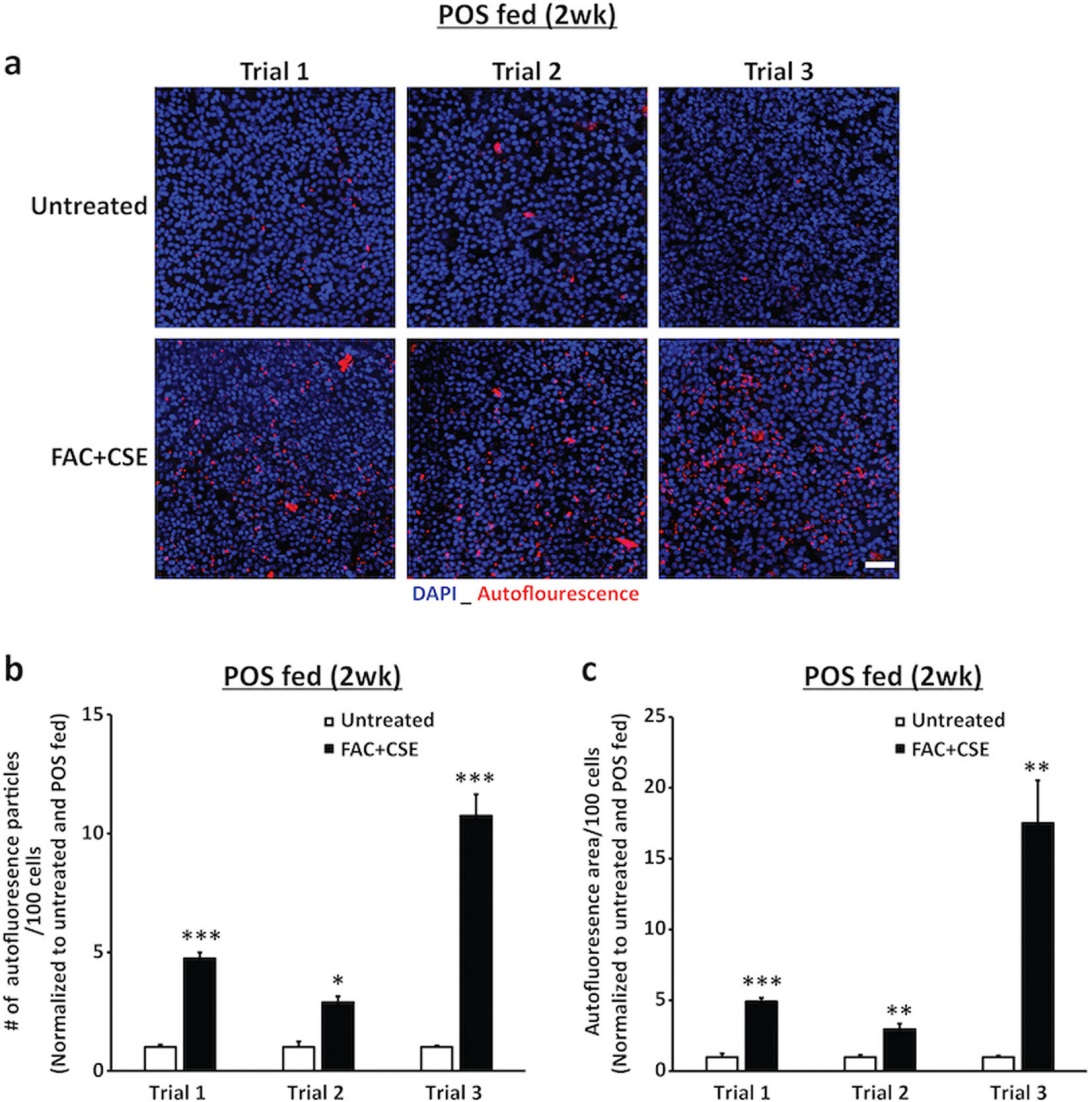
Chronic supplementation of FAC and CSE to culture media over 2 weeks leads to both increased number and area of autofluorescent particles in daily POS-fed hiPSC-RPE cultures. **a** Representative confocal images from three distinct trials showing increased accumulation of autofluorescent particles (red; spectral wavelength ex: 546 nm, em: 560–615 nm) in FAC + CSE-treated (200 µg/ml + 0.5%) and daily POS-fed (20 POS/RPE/cell) hiPSC-RPE cultures compared to untreated but daily POS-fed hiPSC-RPE cultures. Of note Hoescht-stained RPE cell nuclei are seen in blue. **b–c** Quantitative analysis of autofluorescence in untreated and POS-fed hiPSC-RPE cultures versus POS-fed FAC + CSE-treated hiPSC-RPE cultures in three distinct trials showed both increased number (**c**) and area (**d**) of autofluorescent particles in POS-fed FAC + CSE-treated hiPSC-RPE cultures in each individual trial. Data are presented as mean ± SEM. ***P ≤ 0.001, **P ≤ 0.01, *P ≤ 0.05. *n* = 3 independent trials

Our previous results had shown that addition of FAC and/or CSE to the cell culture media for short duration (24 h) does not adversely impact hiPSC-RPE morphology, TER and cell viability (Figs. 2–4 and Supplementary Fig. 1). However, before evaluating the impact of FAC + CSE treatment on autofluorescent material accumulation after daily POS feeding for 2 weeks, we wanted to ensure that simultaneous stress of daily POS feeding and FAC + CSE exposure together for chronic duration (2 weeks) was not toxic to the cells. In these experiments, parallel cultures of hiPSC-RPE cells were either designated as (i) “untreated cultures” and fed daily with only a physiologic dose of POS (~20 POS per RPE cell per day) or (ii) “FAC + CSE-treated cultures” and were simultaneously exposed to both POS and FAC + CSE on a daily basis. Notably, collective stress of chronic POS feeding in conjunction with FAC and CSE exposure, was not detrimental to hiPSC-RPE viability or tight junction integrity as evaluated by live-cell staining (Fig. 5a), ZO-1 staining (Fig. 5b) and TER measurements (Fig. 5c). We next investigated whether continuous exposure to excess Fe and/or cigarette smoke over a chronic (2 weeks) timeframe exacerbated autofluorescent material (POS digestion products) accumulation in hiPSC-RPE cells. Specifically, autofluorescence accumulation (ex: 546 nm, em: 560–615 nm) consistent with lipofuscin accumulation^50,51^ was compared to POS-fed untreated versus POS-fed FAC + CSE-treated hiPSC-RPE cultures. Confocal microscopy analyses of autofluorescence levels revealed increased accumulation of autofluorescent material in POS-fed FAC + CSE-treated hiPSC-RPE compared to only POS-fed untreated hiPSC-RPE cultures (Figs. 5d and 6a–c and Supplementary Table 2). Specifically, quantitative analyses of autofluorescence levels in three distinct trials revealed both (i) a greater number of autofluorescent particles per 100 RPE cell nuclei (Fig. 6a, b and Supplementary Table 2) and (ii) an increased area of autofluorescence per 100 RPE cell nuclei in POS-fed FAC + CSE-treated hiPSC-RPE cultures when compared to untreated hiPSC-RPE cultures (Fig. 6a, c and Supplementary Table 2). Of note, utilizing orthogonal view of autofluorescence in RPE whole mounts immuno-stained for cell nuclei (Hoechst) and tight junction protein (ZO-1), we also confirmed the intracellular pattern of autofluorescent material accumulation in both POS-fed and untreated and POS-fed and FAC + CSE-treated hiPSC-RPE cultures (Fig. 5d, bottom panel). Altogether, these results demonstrate that in the presence of a physiologically relevant stressor, daily POS feeding, exposure of hiPSC-RPE cultures to AMD-relevant stressors, excess Fe, and cigarette smoke, is sufficient to cause increased autofluorescent material accumulation, a known pathological hallmark linked to AMD development^50^.

## Discussion

RPE cell dysfunction in the retina plays a central role in several retinal degenerative diseases, including AMD^52–54^. Although inhaled smoke and altered Fe metabolism have been implicated in AMD development and progression^2,4,7,8,47^, the precise impact of cigarette smoke and/or excess Fe on human RPE cells and its consequence for the development of AMD-relevant pathological changes have not been established. In this study, we utilized FAC and CSE to mimic Fe and cigarette smoke exposure in hiPSC-RPE cells and showed that, in the absence of any genetic defect, these AMD-associated risk factors can alter a key RPE function, phagocytosis and degradation of shed POS, and subsequently lead to increased accumulation of autofluorescent material within the RPE cells. Specifically, our data show a stressor-specific impact of FAC and/or CSE on POS processing in hiPSC-RPE cells with (i) reduction in POS uptake after acute exposure to FAC or FAC + CSE (Figs. 2d, e and 4a, b) and (ii) decrease in POS degradation following combined exposure to FAC + CSE (Fig. 4a, c). Notably, reduced POS degradation in FAC + CSE-treated hiPSC-RPE cultures correlates with decreased levels of active CTSD (Fig. 4d, e), a lysosomal enzyme involved in POS digestion. Furthermore, consistent with impaired POS degradation, chronic exposure (2 weeks) to FAC + CSE in the presence of daily POS feeding is sufficient to elicit increased accumulation of autofluorescent material (plausibly POS digestion products), in hiPSC-RPE cells (Figs. 5, 6).

In numerous retinal degenerative diseases that affect the outer retina, impairment of a specific RPE function, phagocytosis and degradation of shed POS contributes to the disease development. For instance, impaired POS uptake by RPE cells is causal in RP, harboring mutations in the *MERTK* gene^38,39^. Similarly, impaired POS processing by RPE cells has been linked to Stargardt disease pathology^55–57^, inherited maculopathies like BD^36,37,58^ and AMD^59,60^. It is noteworthy that a commonality in the pathology of these distinct diseases is the accumulation of autofluorescent material, lipofuscin (POS-breakdown products), in the retina/RPE layer of affected patient eyes. Apart from genetic defects, aging, the single biggest risk factor associated with AMD development, supports increased accumulation of autofluorescent POS-breakdown products within the RPE cells^50^. In fact, aging-associated increases in RPE autofluorescence have been linked to RPE cell death^24^ and development of AMD pathology^50^. Interestingly, the RPE cell monolayer is likely to be exposed to both Fe overload and cigarette smoke with aging. In support, aging-associated increases in RPE Fe levels have been reported in both rodent models^61^ and human subjects^8,9^. Furthermore, cigarette smoke exposure is a lifestyle risk factor that is more prevalent in adults, increasing with age in the age group of 18–64 years^19^. Notably, Fe and cigarette smoke have been shown to affect cellular compartments/pathways involved in POS processing by RPE cells^6,29,30,32^. Taken in the context of our results showing combined Fe and cigarette smoke exposure in control hiPSC-RPE is sufficient to reduce active-CTSD levels, impair POS digestion and cause increased autofluorescent material accumulation, these data raise questions about the contribution of cell intrinsic (e.g., Fe overload) and extrinsic (e.g., cigarette smoke) stressors in the increased autofluorescence seen with aging in the RPE monolayer in vivo^53^.

It is plausible that changes in POS processing and autofluorescence accumulation observed in our study after FAC + CSE exposure were aided by the fact that hiPSC-RPE cells can be cultured over an extended period of time and this allowed us to use “aged” (~60–90 days in culture) hiPSC-RPE cells in our experiments. This is especially relevant given the fact that in other disease modeling studies, “aging” of hiPSC-RPE cells plausibly contributed to the development of specific human disease phenotype(s), like drusen formation^34^ and autofluorescent material accumulation in culture^36^. Another advantage of hiPSC-based disease modeling utilized in the current study was the ability to select (i) hiPSCs/human subjects and (ii) hiPSC-derived target cell fitting the specified criteria. To evaluate the singular role of Fe and/or cigarette smoke in RPE cell function specifically, we chose control hiPSC-RPE lines that did not harbor a known gene-defect making them susceptible to MD. Furthermore, the ability to use multiple (*n* ≥ 3) hiPSC control lines allowed us to introduce biologic variability in a cell culture model assessing the impact of extrinsic stressors on RPE phagocytosis and degradation. On the flip side, we did not pursue the previously shown consequences of autofluorescence accumulation (e.g., RPE cell death^62^, increased oxidative stress^63^) in hiPSC-RPE cultures. This would be an important consideration for future studies, especially given the fact that presence of Fe overload and/or cigarette smoke exposure could interact with and further exacerbate the consequences of increased autofluorescent POS-digestion by-products in RPE cells. For instance, a recent study has shown a role of Fe in POS-mediated RPE dysfunction through Fe interactions with bisretinoids in lipofuscin supporting RPE cell damage^64^. The long-term impact of Fe and cigarette smoke on POS uptake and digestion is also of future interest in that we limited our FAC/CSE/FAC + CSE exposure to acute (24 h) experiments when evaluating the rate of POS degradation. It is plausible that extended exposure to FAC or CSE by itself would have been sufficient to reduce active-CTSD levels and decrease POS degradation and thereby increase autofluorescent material accumulation in hiPSC-RPE cells. However, utilizing FAC, CSE, and FAC + CSE supplementation over acute (24 h) timeframe, helped highlight potentially more detrimental consequences of multiple simultaneous environmental stressors, a logical but yet untested scenario with regard to RPE cell dysfunction and AMD development.

In summary, we report that excess Fe and cigarette smoke alone and in combination can impair POS processing plausibly by affecting lysosomal function and lead to increased autofluorescent material accumulation in control hiPSC-RPE. This has important implications for the causative role of cell intrinsic and extrinsic stressors in instigating RPE cell dysfunction in retinal degenerative diseases, like AMD.

## Materials and methods

### Generation and characterization of hiPSCs

Collection of patient samples and subsequent experimental analyses were performed in accordance with University of Rochester Institutional Regulatory Board approval (RSRB00056538) and conformed to the requirements of the National Institutes of Health and Declaration of Helsinki. Skin fibroblasts from individuals with no known history of retinal disease were reprogrammed into hiPSCs lines using a previously described protocol^34,65^. Briefly, fibroblasts were maintained in fibroblast medium containing DMEM with high glucose, 10% fetal bovine serum, GlutaMAX^TM^ and 100 U/mL penicillin-streptomycin (Thermo Fisher Scientific, Waltham, MA, USA). iPSCs were generated by reprogramming the fibroblasts with nonintegrating episomal vectors containing six reprogramming factors (OCT-4, SOX2, KLF4, L-MYC, LIN28, and shRNA for p53) as described previously^65^. Reprogramming was initiated by using the nucleofection kit for primary fibroblasts (Lonza) and nucleofected using the Nucleofector 2b Device (Lonza, Program T-016) by transfecting 60,000 fibroblasts with 1 µg each of the following plasmids: pCXLE-hOCT4-shP53, pCXLE-hSK and pCXLE-hUL (Addgene plasmids #27077, 27078, 27080). Transfected cells were then maintained and grown in fibroblast medium for 6 more days following trypsinization and plating 1 × 10^5^ cells onto a 10 cm dish that was previously seeded with irradiated mouse embryonic fibroblast (MEF) feeder layer (MTI-GlobalStem, Thermo Fisher Scientific). The following day, the medium was changed to hiPSC medium (DMEM/F12 with 20% Knockout serum replacement, 1% MEM-NEAA, 1% GlutaMAX^TM^ and 100 ng/mL FGF2). At days 17–30 from transfection, iPSC colonies started appearing and were manually picked and expanded for further characterization. Multiple hiPSC clones were generated, isolated and characterized for expression of pluripotency markers (OCT3/4, NANOG) for every control fibroblast sample by immunocytochemical analysis as previously described^34,36,66,67^. The reprogrammed iPSC lines were maintained undifferentiated on Matrigel^®^ (Corning, Corning, NY, USA) and supplemented with mTESR^TM^1 (STEMCELL Technologies, Vancouver, Canada) or maintained on MEFs and supplemented with hiPSC medium. For all hiPSC lines utilized in this study, pluripotency characterization has been previously published^34,67^.

### Differentiation and culture of hiPSC-RPE

hiPSCs were grown either on MEF feeder layer or on Matrigel^®^ as described previously^68,69^. Differentiation of hiPSCs to obtain RPE was carried out using previously established protocols^69,70^. RPE cells in these adherent differentiated hiPSC cultures^68^ were allowed to mature for a total of 60–90 days prior to passaging and plating onto nonpermeable plastic support (24-well plates) to yield passage 1 (P1) hiPSC-RPE monolayers^36,68^. Mature monolayers of P1 hiPSC-RPE on 24-well plates were terminally passaged onto either Transwell^™^ inserts (CoStar, Corning) or 24-well or 6-well plates using the above described protocol to obtain mature monolayers of P2 hiPSC-RPE cells^36,68^. Unless stated otherwise, parallel age-matched hiPSC-RPE monolayers on transwells and/or 24- and 6-well plates at P2 were used in all experiments.

### Preparation and characterization of cigarette smoke extract

CSE was prepared by bubbling smoke into 20 ml of serum-free RDM using 1R3F research grade cigarettes (Kentucky Tobacco Research Council, Lexington, KT, USA)^71,72^. The optical density of freshly prepared CSE was measured at a wavelength of 320 nm to characterize and normalize its strength. Of note, an optical density of 0.65 is considered to represent 100% CSE^71^. Furthermore, CSE was prepared fresh daily for single use, sterile filtered using 0.22 µm Millex syringe filters (EMD Millipore, Billerica, MA, USA) and diluted to the desired concentration in serum-free RPE cell culture maintenance media (RDM).

### Acute and chronic supplementation of hiPSC-RPE cultures with FAC and CSE

The amount of FAC and CSE utilized in this study to assess the acute (24 h, FAC at 200 µg/ml, CSE at 0.5%) and chronic (2 weeks to 1 month, FAC at 50 and 200 µg/ml, CSE at 0.5%) impact of excess Fe and CSE was based on previously published studies in primary RPE cell cultures (mice and human fetal RPE), and immortalized cell lines (HepG2 and ARPE-19)^5,41–44^. Specifically, hiPSC-RPE cultures were supplemented with FAC (50 and 200 μg/ml), CSE (0.5%), or FAC + CSE (200 μg/ml + 0.5%) in the cell culture maintenance media (RDM) in acute (24 h) and chronic (2 weeks to 1 month) experiments. Of note, hiPSC-RPE cells from parallel cultures serving as untreated controls were fed the same amount of RDM alone on a daily basis. In chronic experiments, lasting 2 weeks to 1 month, media of untreated hiPSC-RPE cultures and FAC and CSE and/or FAC + CSE-treated hiPSC-RPE cultures were replaced on a daily basis. Furthermore, in experiments evaluating phagocytosis and degradation of POS and autofluorescence accumulation post-POS feeding, untreated hiPSC-RPE cultures were fed RDM and POS (20 POS/RPE/day) whereas treated hiPSC-RPE cultures were fed RDM supplemented with FAC, CSE, and FAC + CSE and the same amount of POS during daily media change. Of note, media change and daily POS feeding in these experiments were performed at the same time each day.

### Measurement of TER

TER of hiPSC-RPE monolayers on transwell inserts was measured using an epithelial volt–ohm meter (EVOM2, World Precision Instruments, Sarasota, FL, USA) following manufacturer’s instructions as previously described^73^. In 24 h acute experiments, TER was measured once at baseline before the hiPSC-RPE cells were subjected to FAC, CSE, and/or FAC + CSE treatments, and at the end of the experiment at the 24 h time point. For chronic experiments, lasting 2 weeks, baseline TER was measured at day 0 prior to the start of treatments and subsequently at day 14. Of note, in accordance with other published studies, TER measurements reported in this study were calculated by multiplying blank subtracted raw TER values with the surface area of the transwell filters^68^.

### Assessment of cell viability

Calcein-AM (Life Technologies, Carlsbad, CA, USA) was used to determine the cell viability in accordance with the manufacturer’s protocol. Specifically, hiPSC-RPE cultures were incubated in the dark with calcein-AM (5 µM) and nuclear staining dye (Hoechst, 1:2000; Life Technologies) for 30 min at 37 °C. Calcein-AM and Hoechst-stained hiPSC-RPE samples were imaged on a Leica DM IRBE inverted microscope (Leica Microsystems, Buffalo Grove, IL, USA) using a Lumenera INFINITY3–1 camera and INFINITY ANALYZE software (Lumenera, Ontario, Canada).

### Prussian blue staining

hiPSC-RPE cultures were incubated with equal parts of 20% aqueous solution of hydrochloric acid (HCl) and 10% aqueous solution of K_4_[Fe(CN)_6_]_4−_ for 20 min. hiPSC-RPE cells were then washed three times with double distilled water (ddH_2_O) for 5 min each and immediately imaged using a Motic moticam2000 color camera (Kowloon, Hong Kong).

### Quantitative real-time PCR

As described previously^34^, total RNA was extracted using the RNeasy Mini Plus Kit and DNase I treatment was performed to digest any residual genomic DNA (Qiagen, Germantown, MD). cDNA was synthesized using the iScript cDNA Synthesis Kit (Bio-Rad, Hercules, CA) following the manufacturer’s instructions. Quantitative real-time PCR (qRT-PCR) experiments were carried out using gene-specific primers (Supplementary Table. 3) and the Sso Advanced SYBR Green Supermix (Bio-Rad) in a Bio-Rad CFX Thermal cycler (40 cycles). Data were analyzed using Bio-Rad CFX software (Bio-Rad) and Microsoft Excel.

### Phagocytosis and degradation of POS

Age-matched mature monolayers of hiPSC-RPE cells on transwell inserts were utilized to evaluate phagocytosis and degradation of POS at 0 h and 24 h respectively using a previously described protocol^36,58^. Briefly, hiPSC-RPE cultures were fed unlabeled POS (20 POS/RPE cell; Invision Bioresources, Seattle, WA, USA) for 2 h. At the end of the 2 h incubation, POS containing media was removed and RPE cells were washed vigorously five times with 1X phosphate buffer saline (PBS) to remove any unbound POS remaining on the RPE cell surface. RPE cells were either immediately harvested (0 h time point) or continued in culture and harvested at the 24 h time point for analyses by western blotting. Specifically, the levels of a POS-specific protein, RHO (1:500, EMD Millipore) relative to loading control ACTN (1:500, Santa Cruz Biotechnology, Dallas, TX, USA) were determined by quantitative western blotting to assess the uptake of POS (0 h) and rate of degradation of ingested POS (24 h). Of note, monomer (~35 kDa), dimer (~70 kDa) and multimer bands (>70–250 kDa) and plausibly glycosylated forms (40 kDa) of RHO were all considered for RHO quantification for western blot analyses. Furthermore, when testing the effect of specific stressors (FAC and/or CSE) on phagocytosis/degradation, hiPSC-RPE cultures were treated with FAC (200 μg/ml) and/or CSE (0.5%) concurrently with POS feeding (20 POS/RPE cell). In addition, when cell culture media were replaced after 2 h POS feeding, the fresh cell culture media were supplemented with the appropriate stressor (FAC, CSE, or FAC + CSE).

### Zymography analyses for MMP-1 activity

MMP-1 activity was analyzed by performing zymography on conditioned media obtained from the basal chamber of hiPSC-RPE cultures on transwells in accordance with a previously described protocol^74^. Briefly, conditioned media samples were electrophoresed on 10% polyacrylamide gels containing 1 mg/ml collagen (Sigma-Aldrich). Subsequently, the polyacrylamide gels were incubated in 2.5% Triton-X-100 in 1X PBS for a period of 2 h. After three additional rinses, the gels were incubated in prewarmed MMP-1 incubation buffer (50 mM Tris-HCl, pH, 7.4, 5 mM CaCl_2_, 0.001% NaN_3_, 0.005% Triton-X-100, pH 7.75) for 36 h at 37 °C. Following a brief rinse in destaining solution (1:3:6 acetic acid:methanol:water), the gel was stained with 0.2% Coomassie blue solution for 2–3 h at room temperature. Finally, the gels were rinsed again in destaining solution and imaged on Azure C500 imaging system (Azure Biosystems, Dublin, CA, USA). Of note, quantitative analyses were carried out using Image Studio Lite Version 5.2 and Microsoft Excel.

### Quantitative western blot analyses

hiPSC-RPE cells were lysed and an equivalent microgram load of protein from untreated and treated samples were resolved on 4–20% Tris-HCl gradient gels (Bio-Rad) and transferred onto low fluorescence polyvinylidene difluoride (PVDF) membranes (Bio-Rad) as previously described^34^ and probed with the following primary antibodies: RHO, ACTN, or CTSD (1:500, Santa Cruz Biotechnology). Secondary antibodies utilized were near-Infrared fluorescent (1:10,000, Licor Biosciences) or peroxidase-conjugated secondary antibodies (1:10,000, Azure Biosystems). The blots were either imaged on Odyssey Infrared Imager (Licor Biosciences) or developed using the Radiance Plus Chemiluminescence Kit and imaged on Azure C500 imaging system (Azure Biosystems). Of note, prior to reprobing with a different antibody, the PVDF membrane was stripped using NewBlot PVDF stripping buffer (Licor Biosciences) for 60 min at room temperature. Quantitative analysis of all the western blotting data was carried out using the image acquisition software (Licor Odyssey 3.0 and/or Image Studio Lite version 5.2) and Microsoft Excel.

### Immunocytochemical analyses

Immunocytochemistry of hiPSC/hiPSC-RPE cultures was performed using a previously published protocol^34,67^. Briefly, cells were fixed in 4% paraformaldehyde for 30 min at 4 °C followed by blocking in 1X blocking buffer [10% normal donkey serum (ImmunoReagents Inc., Raleigh, NC, USA) and 0.1% Triton-X-100 in 1X PBS] for 1 h. This was followed by incubation in a primary antibody solution with 0.5X blocking buffer at 4 °C overnight. The samples were washed two times in 0.05% Triton-X-100 in 1X PBS the following day and incubated in host-specific secondary antibodies diluted with 0.5X blocking buffer for 1 h at room temperature. Samples were then washed twice in 0.05% Triton-X-100 in 1X PBS, incubated with nuclear staining dyes (DAPI or Hoechst; Life Technologies) for 15 min in PBS and coverslipped in Prolong gold (Life Technologies). Of note, primary antibodies used for immunocytochemical analysis included EZR (1:100, Cell Signaling Technology, Danvers, MA) and ZO-1 (1:100, Life Technologies). Secondary antibodies used in this study were Alexa Fluor-conjugated (Life Technologies) at a concentration of 1:500. Image acquisition was carried on a confocal microscope (LSM 510 META, ZEISS, Thornwood, NY, USA) using Zen 2009 software (ZEISS).

### Quantification of autofluorescence in POS-treated cultures

hiPSC-RPE cultures post chronic (2 weeks) POS feeding were washed five times with 1X PBS to remove any unbound POS remaining on the RPE cell surface. Subsequently immunocytochemical analyses (as described above) were performed to localize ZO-1 and DAPI. After image acquisition, autofluorescence in excitation (543 nm) and emission (560–615 nm) spectra consistent with lipofuscin were evaluated in *n* = 6 random images per sample per trial using ImageJ software (NIH) and plotted as (i) the number of autofluorescence particles and (ii) total autofluorescent area per 100 cells (as measured by DAPI-stained nuclei).

### Statistics

All experiments were performed on at least *n* ≥ 3 hiPSC-RPE cultures obtained from independent hiPSC-differentiation runs and data throughout the manuscript are expressed as mean ± SEM. Furthermore, statistical significance (*P* ≤ 0.05) was evaluated using an unpaired two-tailed Student’s t-test in Microsoft Excel.

## Supplementary information

Supplementary Figure 1

Supplementary Figure 2

Supplementary Figure 3

Supplementary Table 1

Supplementary Table 2

Supplementary Table 3

Supplemental Material File #1

## Abbreviations

RPE, Retinal pigment epithelium; POS, Photoreceptor outer segments; AMD, Age-related macular degeneration; hiPSC, Human induced pluripotent stem cell; CSE, Cigarette smoke extract; RP, Retinitis pigmentosa; B(a)P, Benzo(a)Pyrene; TER, Transepithelial resistance; SFD, Sorsby’s fundus dystrophy; DHRD, Doyne honeycomb retinal dystrophy; FAC, Ferric ammonium citrate; RDM, Retinal differentiation media.
