# Supplementary figures and images for: Environmental stress impairs photoreceptor outer segment (POS) phagocytosis and degradation and induces autofluorescent material accumulation in hiPSC-RPE cells
Source: Cell Death Discov. 2019 May 16;5:96. doi: 10.1038/s41420-019-0171-9 (PMC6522536; doi:10.1038/s41420-019-0171-9)

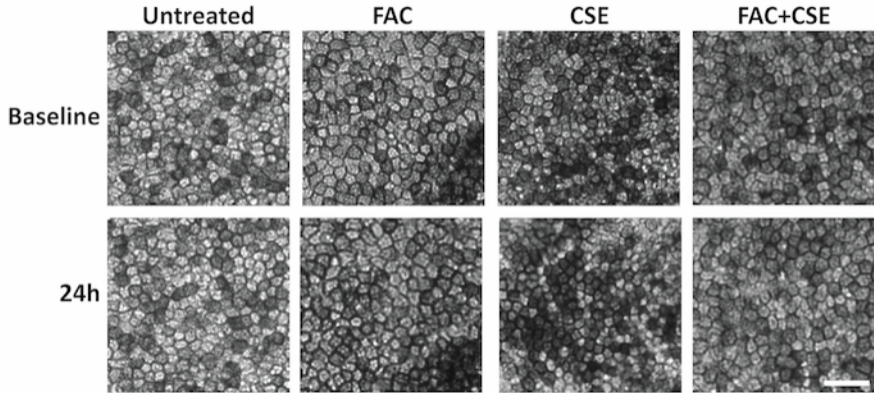

Supplement: Supplementary file 1 — Supplementary Figure 1 [file 41420_2019_171_MOESM1_ESM.pdf]

**a**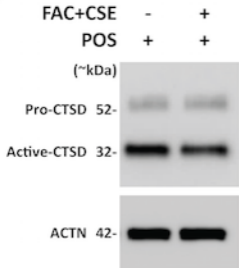**b**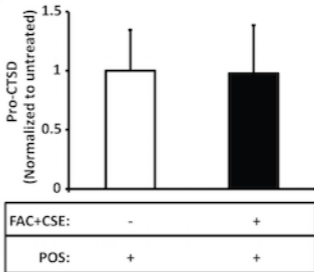

Supplement: Supplementary file 2 — Supplementary Figure 2 [file 41420_2019_171_MOESM2_ESM.pdf]

**a**

FAC      -      +  
POS      +      +

(~kDa)

Pro-CTSD 52-

Active-CTSD 32-

ACTN 42-

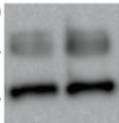**b**

Active-CTSD  
(Normalized to untreated)

1.5  
1.0  
0.5  
0

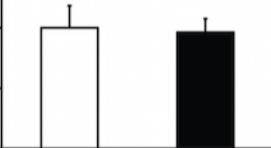

FAC:

-

+

POS:

+

+

Supplement: Supplementary file 3 — Supplementary Figure 3 [file 41420_2019_171_MOESM3_ESM.pdf]
