# Supplementary material for: Environmental stress impairs photoreceptor outer segment (POS) phagocytosis and degradation and induces autofluorescent material accumulation in hiPSC-RPE cells
Source: Cell Death Discov. 2019 May 16;5:96. doi: 10.1038/s41420-019-0171-9 (PMC6522536; doi:10.1038/s41420-019-0171-9)
Supplement: Supplementary file 4 — Supplementary Table 1 [file 41420_2019_171_MOESM4_ESM.docx]

| **Gene** | **Untreated** | **FAC 50 µg/ml** |
| --- | --- | --- |
| *CP* | 1 ± 0.12 | 1.92 ± 0.99* |
| *HFE* | 1 ± 0.20 | 10.97 ± 3.93*** |
| *GSS* | 1 ± 0.11 | 2.15 ± 1.11* |
| *TF* | 1 ± 0.16 | 2.05 ± 0.76* |

**Supplementary Table 1.** Relative expression of specific iron-regulatory genes in untreated vs. FAC-treated (50 µg/ml, ~1mo) hiPSC-RPE cells. Gene expression was evaluated relative to loading control, GAPDH, and subsequently normalized to untreated hiPSC-RPE cells. Data are presented as mean ± SEM, n= 3 independent trials. **P* ≤ 0.05, ***P* ≤ 0.01 and ****P* ≤ 0.001
