# Supplementary material for: Environmental stress impairs photoreceptor outer segment (POS) phagocytosis and degradation and induces autofluorescent material accumulation in hiPSC-RPE cells
Source: Cell Death Discov. 2019 May 16;5:96. doi: 10.1038/s41420-019-0171-9 (PMC6522536; doi:10.1038/s41420-019-0171-9)
Supplement: Supplementary file 5 — Supplementary Table 2 [file 41420_2019_171_MOESM5_ESM.docx]

**Supplementary Table 2**. Quantitative analyses of autofluorescent particles in parallel cultures of FAC+CSE-treated (200 µg/ml+0.5%; 2wk) and POS-fed (20 POS/RPE cell/day) hiPSC-RPE cells and untreated but POS-fed (20 POS/RPE cell/day) hiPSC-RPE cells. Data are presented as mean ± SEM, n=3 independent trials. **P* ≤ 0.05, ***P* ≤ 0.01 and ****P* ≤ 0.001.

**POS fed (2wk)**

|  | **Autofluoresence/100 cells** | | **Autofluoresence area/100 cells** | |
| --- | --- | --- | --- | --- |
|  | Untreated | FAC+CSE | Untreated | FAC+CSE |
| Trial 1 | 1 ± 0.11 | 4.74 ± 0.25*** | 1 ± 0.27 | 4.93 ± 0.26*** |
| Trial 2 | 1 ± 0.24 | 2.89 ± 0.25* | 1 ± 0.16 | 2.97 ± 0.40** |
| Trial 3 | 1 ± 0.06 | 10.75 ± 0.90*** | 1 ± 0.10 | 17.52 ± 3.01** |
