# Supplementary material for: Environmental stress impairs photoreceptor outer segment (POS) phagocytosis and degradation and induces autofluorescent material accumulation in hiPSC-RPE cells
Source: Cell Death Discov. 2019 May 16;5:96. doi: 10.1038/s41420-019-0171-9 (PMC6522536; doi:10.1038/s41420-019-0171-9)
Supplement: Supplementary file 6 — Supplementary Table 3 [file 41420_2019_171_MOESM6_ESM.docx]

| **Primer name** | **Primer Sequence (5'-3')** |
| --- | --- |
| CP FOR  CP REV | TATTCTCCTGGGTGCTCCTCAA  ATACCTTGCCCATTCCCTCTG |
| HFE FOR  HFE REV | CAACAAGTGCCTCCTTTGGT  GGGGGTACAGCCAAGGTTAT |
| GSS FOR  GSS REV | AGGCGAACTAGTGTTGGGAT  AGAGCGTGAATGGGGCATAG |
| TF FOR  TF REV | GGTGGCAGAGTTCTATGGGTC  ACAGTAAAGTAAGCCTATGGGGA |
| GAPDH FOR  GAPDH REV | AGCAAGAGCACAAGAGGAAGAG  GAGCACAGGGTACTTTATTGATGG |

**Supplementary Table 3.** The nucleotide (5’-3’) sequence of forward and reverse primers used in quantitative real-time PCR analyses.
