# Supplementary material for: Environmental stress impairs photoreceptor outer segment (POS) phagocytosis and degradation and induces autofluorescent material accumulation in hiPSC-RPE cells
Source: Cell Death Discov. 2019 May 16;5:96. doi: 10.1038/s41420-019-0171-9 (PMC6522536; doi:10.1038/s41420-019-0171-9)
Supplement: Supplementary file 7 — Supplemental Material File #1 [file 41420_2019_171_MOESM7_ESM.docx]

**Supplemental Material**

**Supplemental Fig 1**. **FAC and/or CSE supplementation of culture media does not affect RPE cell morphology in both unfed and POS-fed cultures**. Representative light microscopy images of hiPSC-RPE showing RPE-characteristic cobblestone morphology of untreated at baseline (top panel) and treated and after acute exposure (24h) to FAC (200 µg/ml), CSE (0.5%), FAC+CSE (200 µg/ml + 0.5%), hiPSC-RPE cultures.

**Supplemental Fig 2. FAC+CSE supplementation for 24h does not affect pro-CTSD expression in hiPSC-RPE cells**. **a and b** Representative Western blot images **(a)** and corresponding quantitative analyses **(b)** showing similar levels of pro-CTSD in POS-fed (20 POS/RPE cell) and FAC+CSE-treated (200 µg/ml + 0.5%, 24h) hiPSC-RPE cultures compared to only POS-fed hiPSC-RPE cultures. Note: ACTN served as a loading control. Data are presented as mean ± SEM, n=3 independent trials.

**Supplemental Fig 3.**  **FAC supplementation for 24h does not affect active-CTSD expression in hiPSC-RPE cells.**  **a and b** Representative Western blot images **(a)** and corresponding quantitative analyses **(b)** displaying no difference in active-CTSD expression between untreated and POS-fed (20 POS/RPE cell) vs. FAC-treated (200 µg/ml, 24h) and POS-fed hiPSC-RPE cells. Note: ACTN served as a loading control. Data are presented as mean ± SEM, n=3 independent trials.
